# Two Complete and One Draft Genome Sequence of Nonproteolytic Clostridium botulinum Type E Strains NCTC 8266, NCTC 8550, and NCTC 11219

**DOI:** 10.1128/genomeA.00083-15

**Published:** 2015-03-12

**Authors:** Charlien Clauwers, Yves Briers, Rob Lavigne, Chris W. Michiels

**Affiliations:** a Laboratory of Food Microbiology, Department of Microbial and Molecular Systems and Leuven Food Science and Nutrition Research Centre (LFoRCe), KU Leuven, Heverlee, Belgium; b Laboratory of Gene Technology, Department of Biosystems, KU Leuven, Heverlee, Belgium

## Abstract

Group II (gII) nonproteolytic Clostridium botulinum strains are a major cause of foodborne botulism outbreaks. Here, we report two complete genome sequences of gII type E strains NCTC 8266 and NCTC 8550 and one draft genome sequence of type E NCTC 11219.

## GENOME ANNOUNCEMENT

The anaerobic sporeformer Clostridium botulinum produces the most potent protein toxin known and can cause infant, wound, and foodborne botulism. C. botulinum is organized in four physiologically and phylogenetically different groups, of which gII consists of nonproteolytic and psychrotrophic strains (1). Due to their ability to grow at refrigeration temperatures, gII strains pose a major safety hazard in mildly processed chilled ready-to-eat foods with extended shelf life. Group II can be further subdivided in type B, E, or F, based on the serotypes of the toxin produced (2).

Here, we focus on gII type E strains, for which only two whole-genome sequences are available to date, C. botulinum E1 strain Beluga (six shotgun sequences; NZ_ACSC01000000) and strain Alaska E43 (whole genome; CP001078). We present the high-quality whole-genome sequences of C. botulinum type E NCTC 8266 and NCTC 8550 and the whole-genome shotgun project of NCTC 11219 consisting of three contigs. Strains NCTC 8266 and 11219 are linked to botulism outbreaks caused by salmon (in 1944 and 1978); NCTC 8550 was isolated at the Pasteur Institute (Paris, France) in 1952.

To obtain high-quality genomic DNA, formaldehyde was used for fixation of cells prior to isolation (3). This step was essential to avoid DNA degradation by the abundant extracellular Dnase activity produced in gII C. botulinum cultures. Paired-end libraries were constructed using the NEBNext Ultra gDNA library prep protocol and analyzed on the Agilent BioAnalyzer (VIB Nucleomics Core, Belgium). Sequencing was performed on an Illumina MiSeq sequencer, yielding 3,795,388, 3,669,738, and 4,733,175 reads of 150 bp for NCTC 8266, 8550, and 11219, respectively (with 157-, 152-, and 187-fold total coverage). Genome assembly was performed using the CLC Genomics Workbench version 7 (CLC Bio, Aarhus, Denmark), applying a combinatorial approach of reference assembly against C. botulinum Alaska E43 (3,659,644 bp), *de novo* assembly of nonassembled reads, and manual editing.

Strains NCTC 8266 and 8550 have an almost identical genome size of 3,611,897 and 3,611,898 bp and are highly similar to strain Alaska (99% identity). The draft genome sequence of NCTC 11219 (3,792,082 bp) differs more from Alaska (93% identity). Gene predictions and annotations with the Prokaryotic Genome Annotation Pipeline (PGAP) identified 3,218 putative genes for NCTC 8266 and 8550, and 3,426 putative genes for NCTC 11219, of which 3,060 and 3,215 are protein-coding, respectively. For all strains, 34 rRNA and 79 tRNA genes were found. PHAST (4) identified two potential prophages in NCTC 8266 and NCTC 8550, of which one appears intact (39.2 kb) and one incomplete (24.4 kb). In contrast, NCTC 11219 encodes five prophage regions, of which three are potentially intact (39.6 kb, 28.4 kb, and 26.1 kb) and two are incomplete (27.8 kb and 13.9 kb).

### Nucleotide sequence accession numbers.

The complete genome sequences have been deposited at DDBJ/ENA/GenBank under the accession numbers CP010520 (NCTC 8266) and CP010521 (NCTC 8550). The whole-genome shotgun project of NCTC 11219 has been deposited under the accession number JXMR00000000. The version described in this paper is version JXMR01000000.
